# Characterization of the *Drosophila* Group Ortholog to the Amino-Terminus of the Alpha-Thalassemia and Mental Retardation X-Linked (ATRX) Vertebrate Protein

**DOI:** 10.1371/journal.pone.0113182

**Published:** 2014-12-01

**Authors:** Brenda López-Falcón, Silvia Meyer-Nava, Benjamín Hernández-Rodríguez, Adam Campos, Daniel Montero, Enrique Rudiño, Martha Vázquez, Mario Zurita, Viviana Valadez-Graham

**Affiliations:** 1 Departamento de Genética del Desarrollo y Fisiología Molecular, Instituto de Biotecnología, Universidad Nacional Autónoma de México, Cuernavaca, Morelos, México; 2 Departamento de Medicina Molecular y Bioprocesos, Instituto de Biotecnología, Universidad Nacional Autónoma de México, Cuernavaca, Morelos, México; Oxford Brookes University, United Kingdom

## Abstract

The human *ATRX* gene encodes hATRX, a chromatin-remodeling protein harboring an helicase/ATPase and ADD domains. The ADD domain has two zinc fingers that bind to histone tails and mediate hATRX binding to chromatin. dAtrx, the putative ATRX homolog in *Drosophila melanogaster*, has a conserved helicase/ATPase domain but lacks the ADD domain. A bioinformatic search of the *Drosophila* genome using the human ADD sequence allowed us to identify the *CG8290* annotated gene, which encodes three ADD harboring- isoforms generated by alternative splicing. This *Drosophila* ADD domain is highly similar in structure and in the amino acids which mediate the histone tail contacts to the ADD domain of hATRX as shown by 3D modeling. Very recently the *CG8290* annotated gene has been named *dadd1*. We show through pull-down and CoIP assays that the products of the *dadd1* gene interact physically with dAtrx_L_ and HP1a and all of them mainly co-localize in the chromocenter, although euchromatic localization can also be observed through the chromosome arms. We confirm through ChIP analyses that these proteins are present *in vivo* in the same heterochromatic regions. The three isoforms are expressed throughout development. Flies carrying transheterozygous combinations of the *dadd1* and *atrx* alleles are semi-viable and have different phenotypes including the appearance of melanotic masses. Interestingly, the dAdd1-b and c isoforms have extra domains, such as MADF, which suggest newly acquired functions of these proteins. These results strongly support that, in *Drosophila*, the *atrx* gene diverged and that the *dadd1*-encoded proteins participate with dAtrx in some cellular functions such as heterochromatin maintenance.

## Introduction

The human *ATRX* gene (*hATRX*) was described approximately 20 years ago as the main gene mutated in ATRX syndrome (Alpha-Thalassemia with mental Retardation X-related). *ATRX* is localized on the X chromosome in position Xq13.1–q21.1. Individuals with mutations in this gene present several phenotypic charateristics that may include mental retardation, craniofacial and urogenital deformities, psychomotor failure and alpha-thalassemia [1]. Since its description, there have been important advances in the characterization of the molecular functions of the protein encoded by this gene. In humans, there are mainly two isoforms named hATRX (289 kDa) and hATRXt (t, from truncated, 200 kDa) that are encoded by this gene [2]. Both proteins contain an amino-terminal domain which is composed of PHD and GATA-like zinc fingers, named ADD after the three proteins that contain this domain (ATRX, DNMT3b and DNMT3L). It was recently demonstrated through different *in vitro* and *in vivo* approaches that this domain recognizes the combination of K9me3 and unmethylated K4 residues of the histone H3 tail [3]. This domain directs the protein mainly to pericentric heterochromatin [4]. Mutations described in patients afflicted with the syndrome mainly affect the important amino acids that form the "pocket" of the ADD domain for the histone H3 tail recognition. The hATRX protein additionally has a helicase/ATPase domain, which classifies it as a member of the SNF2 subfamily of chromatin remodelers [5]. The hATRX SNF2 domain has *in vitro* ATPase activity, which can be stimulated both by DNA and nucleosomes [6]. About 50% of the mutations described in patients fall in the ADD domain, whereas the other 50% affect the SNF2 helicase/ATPAse and other protein domains [7]. hATRX, as many chromatin remodelers, has been identified as a component of a complex that includes the histone variant H3.3 chaperone DAXX (Death domain Associated protein). This particular histone variant is incorporated at different chromatin regions, such as promoters, enhancers and heterochromatic regions, and it has been proposed to have dual functions in promoting both an active chromatin state and the maintenance of heterochromatin [8]. hATRX ATPase activity is important for incorporation of the histone variant H3.3 by the chaperone DAXX into specific regions of the chromosomes, such as telomeres and pericentric heterochromatin [9].

Genome-wide studies have identified hATRX as a protein that is able to bind to DNA regions that can acquire a G4 structure conformation, such as telomeres and repetitive G-rich regions [9]; however, the important domain that mediates this interaction it is not yet known.

It is clear though that both the ADD and helicase/ATPase domains play crucial roles during development [1]. The *ATRX* gene is highly conserved through eukaryotic evolution, but in invertebrates and particularly in *Drosophila*, it encodes proteins that lack the ADD N-terminal domain, i.e., the dAtrx_L_ and dAtrx_s_ isoforms are encoded by the same gene but lack an ADD domain. The dAtrx_L_ isoform interacts with HP1a and is localized to heterochromatin, whereas the dAtrx_s_ isoform is localized to euchromatin [10].

We decided to determine if there was a gene in *Drosophila* that could encode proteins with the conserved the amino-terminus of the vertebrate ATRX. Performing *in silico* analyses, we demonstrate that the annotated gene *CG8290*, recently named *dadd1* [11], encodes three proteins with a conserved ADD domain that physically interact with dAtrx_L_. Using a genetic approach, we found that these proteins have important functions during *Drosophila* development and that they cooperate with dAtrx_L_ in certain functions. The evidence leads us to propose that the dAdd1 proteins are orthologs of the amino-terminus of the ATRX protein in vertebrates.

## Materials and Methods

### Ethics statement

All animal handling was approved by the Instituto de Biotecnología Bioethics Comittee, Permit Number 344 (2011/02/10), which follows NOM-062 animal welfare mexican law. All efforts were made to minimize animal suffering. Animals were sacrificed by CO_2_ euthanasia.

### Protein domain structure illustration

**Figure 1:**
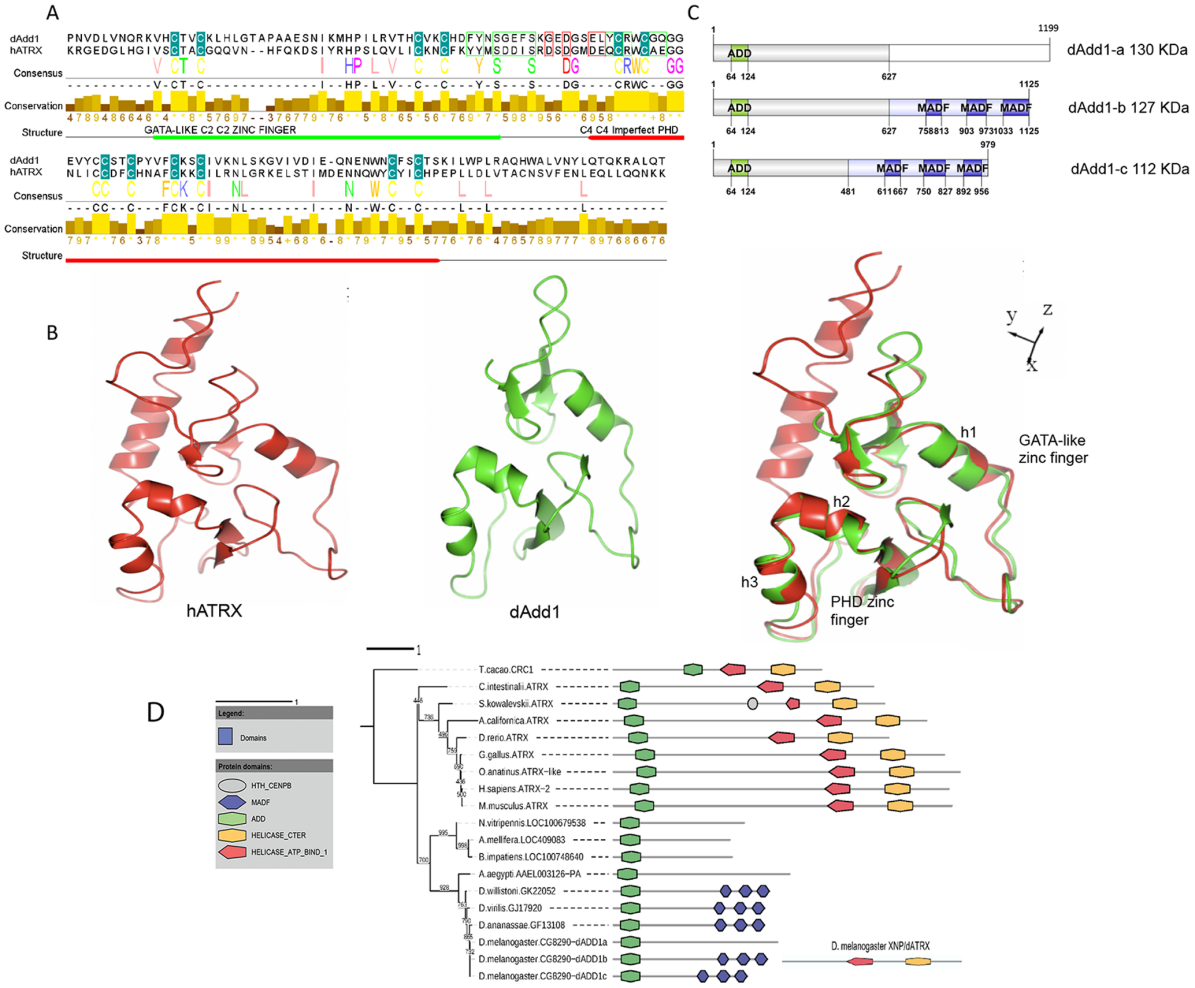
The ADD domain of the hATRX protein is conserved in the dAdd1 proteins of *Drosophila*. A. ADD domain prediction in the protein sequence alignment of hATRX and dAdd1 proteins. Conserved cysteines are shown as yellow letters within a cyan box. The amino acids involved in H3K9me3 and H3K4me0 recognition are marked by green and red boxes respectively. The GATA-like C2-C2 zinc finger and the C4-C4 imperfect PHD are marked by green and red boxes respectively. B. Ribbon representation of ADD domain of hATRX on red (left) and dAdd on green (center) CPH models-3.0 server was used to create the model of the ADD domain of dAdd1. Structural superposition of ADD domains of hATRX and dAdd1 (right). C. Domain organization of dAdd1 (a-c) isoforms. All of them have an N-terminal ADD domain. A C-terminal MADF domain is present in two (dAdd1-b, and dAdd1-c isoforms) copies. D. Maximum Likelihood Phylogenetic Analysis of the ADD domain and the corresponding Protein Domain Architecture information of the containing proteins as computed by PhyML [16] and ScanProsite [19], respectively. The numbers shown represent bootstrap values. Note that the ADD harboring protein underwent a gene fission event during the evolution of insects. Also note that the homologous proteins within the *Drosophila* genus have acquired a tandem MADF domain during its divergence from other insects. This domain is likely to be functional given its conservation within the genus. For the parameters used, refer to Material and Methods.

The domain organization of dAdd1 isoforms (Fig. 1C) and the representation of the fragments assayed by pull-down were performed using the DOG 1.0 Illustrator of Protein Domain Structures [12]. For gene representation we used FancyGene [13].

### Alignments and phylogenetic inference analyses

Multiple alignments were performed with CLUSTALX2 2.1 [14] and the parameters for the Phylogenetic Inferences were used as estimated by ProtTest 2.4 program [15] for selection of models of protein evolution. The Maximum Likelihood Phylogenetic Analysis was computed by PhyML 3.0 [16] with the parameters: Substitution model: WAG (ADD) & LG (Helicase/ATPase) [17]; Bootstrap: 1000; Proportion of invariable sites: 0.12 (Both); Gamma shape parameter: 2.15 (ADD) & 0.81 (Helicase/ATPase). The tree was edited using Interactive Tree Of Life (iTOL) v2 [18] with Protein Domain Architecture information of the containing proteins as predicted by ScanProsite [19].

### Protein structure homology model

In an attempt to determine a protein-modeling of ADD domain (101 amino acid residues), we generated by SWISS-MODEL [20], a three-dimensional structural model of a protein target, based on identity sequence related with structures deposited in the Protein Data Bank. Basically the steps used in homology modeling are the following: template identification, amino acid sequence alignment, model building and model verification (model quality) [21]. The crystal structure of the transcriptional regulator ATRX from *Homo sapiens* [22] PDB entry 3qla chain A, was used as a template for homology modeling. The identity between these two proteins was 37% and the structural similarity was 0.42. It is important to mention that the procedures implemented in SWISS-MODEL allow the modeling of sequences which share at least 35% identity with a known three dimensional structure. Although the resulting models do not represent the real 3D structure, it is accurate enough to learn about the general topology and a possible residue arrangement of the ADD protein sequence.

### Fly stocks

The wild-type flies used in this study were Oregon R (*OreR*) or *w^1118^*, and fly stocks were maintained at 25°C with standard food. The stocks that carried the *atrx* alleles were obtained from the Bloomington Stock Center. The stocks that carried *dadd1* and *Su(var)2-5* alleles were obtained from the *Drosophila* Genetic Resource Center (DGRC), Kyoto Institute of Technology. The *xnp/atrx* alleles (called *atrx* for simplicity) were described by Bassett AR, *et al*., (2008) [10]. The *dadd1* alleles: *dadd1^NP0793^* (w*;P{GawB}^NP0793^/CyO) and *dadd1^NP1240^* (y*w*;P{GawB}^NP1240^/CyO, P{UAS lacZ.UW14} UW14), carry a *P*-element insertion at -225 and -223 bp from the translation initiation codon of the *dadd1* gene, respectively. The *Su(var)2-5^2^* allele is a missense mutation that has been characterized molecularly by Eissenberg *et al*., (1992) [23]. This is a single mutation in the open reading frame: a G-A transition in the first nucleotide of codon 26, resulting in the substitution of methionine for valine that affects the chromodomain. The *Su(var)2-5^5^* allele is an X-ray induced mutation, in which only the first 10 amino acids of HP1a are translated [23]. The position effect variegation (PEV) reporter line BL1 is an inversion allele of the *hsp70-lacZ* transgenic reporter, with the reporter gene positioned adjacent to a 3L pericentric heterochromatin mass [24].

### Genetic crosses

All stocks were outcrossed with *w^1118^*; *Sp/CyO; TM6B, Tb/MKRS* flies for five generations. Chromosomes with the alleles of interest were followed by segregation with specific balancer chromosomes. To reassure the presence of the atrx alleles in these lines, females carrying alleles *atrx^1^, atrx^2^ and atrx^3^* chromosomes were outcrossed with males from parental *atrx^1^* allele and viability was assayed and compared to the previously reported viability [10]. The stocks that carried the *atrx* alleles were established and balanced with *TM6B, Tb* for chromosome 3. The stocks that carried the *dadd1* alleles were established and balanced with the *CyO* balancer for chromosome 2 and followed by *white* complementation. Fly crosses were performed according to standard procedures, three biological replicates were performed. At least 100 flies were examined for each genotype.

### Antibodies

The dAtrx_L_ antibody was previously described [25]. All antibodies were generated by New England Peptide (NEP). The pan-dAdd1 antibody was generated using the peptide: QGGEVYCCSTCPYVFCKSC wich recognizes dAdd1-a, b and c isoforms. For the dAdd1-a isoform, the CDLIKALGSPSVLP peptide was used and for the dAdd1-b isoform the CDKQFCQQLVLAM peptide was used. Specificity of these antibodies was assayed by their capability to recognize dAdd1-a or dAdd1-b fused to GST (see pull-down section and antibody specificity assays section for details) by western blot (Fig. S1). The HP1a antibody was obtained from the Developmental Studies Hybridoma Bank DSHB at the University of Iowa (C1A9). The HA antibody was obtained from Roche (Ref.11867423001). Antibodies used for mock immunoprecipitations were purified IgG from mouse GenScript (Cat. A01007); and purified IgG from rabbit Invitrogen (Cat.02-6102).

#### Antibody specificity assays

To test the specificity of the pan-dAdd1 antibody, a Western blot was performed with 100 µg of S2R+ protein extracts either using as primary antibody the non-depleted pan-dAdd1 antibody fraction or a supernatant from where the pan-dAdd1 antibodies were depleted (Fig. S1A). Depletion was performed by incubating the pan-dAdd1 antibody in PBS, Tween 0.01%, 5% nonfat milk for 2 hours in the presence of the dAdd1-GST fusion protein blotted onto a nitrocellulose membrane, after the incubation period, supernatant fraction was saved (depleted fraction). The GST-dAdd1 fusion protein harbors the dAdd1 QGGEVYCCSTCPYVFCKSC peptide (aa 122-137) that was used to raise the pan-dAdd1 antibody.

To test the specificity of the dAdd1-a and dAdd1-b antibodies, GST-dAdd1-a or b fusion proteins were expressed in bacteria, blotted onto a nitrocelulose membrane and incubated with either anti-dAdd1-a (Fig. S1B) or anti-dAdd1-b (Fig. S1C) showing that the different antibodies recognize their specific substrate. Detection was performed with the PIERCE quimioluminiscence substrate.

### Immunostaining of polytene chromosomes

Polytene chromosomes for immunostaining were obtained from wild type *OreR* or *w^1118^* lines or from *Xnp^Scer\UAS.T:Ivir\HA1^/Sgs3-GAL4* (*w^1118^; P{w^+mC = Sgs3-GAL4.PD^}TP1*) larvae. The *Xnp^Scer\UAS.T:Ivir\HA1^* allele is described in [10], and the *Sgs3-GAL4* (*w^1118^; P{w^+mC = Sgs3-GAL4.PD^}TP1* driver line was used to direct Xnp/dAtrx (called dAtrx for simplicity throughout this manuscript) expression to salivary glands from third instar larvae. Both latter lines were obtained from the Bloomington *Drosophila* Stock Center.

Immunostaining of polytene chromosomes was performed with slight modifications of the protocol described in [25]. Salivary glands from third instar larvae were fixed in solution I (PBS, 3.7% paraformaldehyde and 1% Triton X-100) and then in solution II (3.7% paraformaldehyde, 50% acetic acid). The chromosomes were spread on poly-L-Lysine coated microscope slides. Anti-HP1a (DSHB) antibody was used at 1∶300, anti-dAtrx_L_ antibody at 1∶100, pan-dAdd1 at 1∶50 and anti-HA (Roche) at 1∶50. Secondary antibodies were Alexa fluor 488 used at 1∶500 and Alexa fluor 568 or 594 used at 1∶100 (Invitrogen). Images were taken on a confocal laser-scanning microscope (Olympus FV1000) at the Laboratoratorio Nacional de Microscopía Avanzada (LNMA, UNAM).

For the double dAdd1/dAtrx immunostaining we followed an epitope tagged version of dAtrx_L_ (dAtrx_L_-HA) because the dAdd1 and dAtrx_L_ antibodies were raised in the same species and could not be used together for this experiment.

### Pull-down assays

We generated fusion proteins of several fragments of dAtrx_L_ and the dAdd1 proteins. All the clones used in this work were nucleotide-sequenced. The *Drosophila* LD28477, LD24316 and LD37351 cDNA (BDGP Gold collection of *Drosophila* Genomics Resource Center) were amplified by PCR and cloned in the *Eco*RI, *Not*I, *Sma*I, *Xho*I or *Sal*I sites of the pGEX-4T1 or pGBKT7 vector (details are available upon request). The dAtrx_L_ and dAdd1 fragments were expressed as GST fusion proteins in a bacterial system. For interaction assays, over-expression of dAtrx_L_ fragments fused to GST in bacteria was induced with 0.4 mM IPTG during 3 h. GST-dAtrx_L_ fragments were purified using glutathione-sepharose (Amersham) according to manufacturer's instructions. The dAdd1 fragments were expressed and labelled with S^35^ using an *in vitro* transcription/translation system (TNT-Quick-Coupled Transcription/Translation System from Promega). Pull-down assays were performed according to the manufacturer's instructions.

### Cell culture transfection and co-immunoprecipitation

The dAdd1-a cDNA was cloned into *Eco*RI/*Not*I sites of pAc5.1/V5-HisA vector (Invitrogen). *Drosophila* S2R+ cells were maintained in Schneider medium with 10% fetal bovine serum and 100 µg streptomycin/0.25 µg amphotericin. Cells were cotransfected with 10 µg of each construction by the calcium method (Invitrogen). Fourty-eight hours after transfection, the cells were collected and lysed. Lysates were clarified by centrifugation at 15.7 g at 4°C. CoIPs were performed as indicated in [26]. For embryo-stage immunoprecipitations: embryo nuclear extracts were prepared as described [27]. The Co-IP was performed as described in [28]. Antibodies used for mock immunoprecipitations were purified IgG from mouse GenScript (Cat. A01007); and purified IgG from rabbit Invitrogen (Cat.02-6102).

### RT–PCR assays

RNA was obtained using the Trizol reagent (Invitrogen®) from embryos (0–3 and 3–21 hour post-fertilization), 1^st^, 2^nd^ and 3^rd^ instar larvae (L1, L2 and L3), pupae (P), pharate (Ph) and female and male adults Oregon R flies (F and M). 10 µg of total RNA was converted to cDNA using reverse transcriptase enzyme (Invitrogen) and oligo-dT and random primers (Stratagene®). To assess the presence of the transcripts the following oligonucleotides were used for the PCR reaction: dAdd1-a forward (5′CATCTTACGGGCAAAGTGGT-3′); dAdd1-a reverse (5′CAGGCTGGCCAATATCGTGG-3′); dAdd1-b forward (5′GCTTGTCATCGGGCATATCT-3′); dAdd1-b reverse (5′GCTCATAAGCAGCCAGCAGT-3′); dAdd1-c forward (5′ACAGCGGCAGCAACGGAAGC-3′); dAdd1-c reverse (5′GCGGAAGTCCTTGCAGCGGT-3′); rp49 forward (5′TCAAGATGACCATCCGCCCA-3′); rp49 reverse (5′GTTCTCTTGAGAACGCAGGC-3′). rp49 was used as an RT- PCR control.

### Chromatin Immunoprecipitation

S2R+ cells or third instar larvae salivary glands from wild type organisms were fixed in 1% formaldehyde. The fixation reaction was stopped by adding glycine (125 mM). Cells or salivary glands were washed and resuspended in lysis buffer, and sonication was performed until the size of chromatin reached between 200 and 800 bp. Pre-clearing, antibody incubations, immunoprecipitation and phenol:chlorophorm extractions were performed as described in [25]. For the ‘mock’ condition, a pre-immune sera against dAtrx or an anti-GFP antibody was used. The following oligonucleotides were used: rover forward (5′-CAACCAAGACCAACCTACCC-3′); rover reverse (5′-GCTCATTTTAGTCTGTCCGC-3′) [29]; for TAS-L, TAS3L_ChIP1 (5′-TGACTGCCTCTCATTCTGTC-3′) and TAS3L_ChIP2 (5′-TATCATCTCGTTCATCCGCC-3′) [30]. qPCRs were performed using the light cycler DNA master SYBR green 1 run in a Lyght cycler 1.5 (ROCHE), and the quantification of %INPUT was performed as in [31].

### β-galactosidase quantitative assay

Detection of β-galactosidase in adult flies was performed as in Gu and Elgin (2013) [32]. For quantitative β-galactosidase assays, flies were homogenized in 300 µl of assay buffer (50 mM potassium phosphate, 1 mM MgCl2, pH 7.5), followed by spinning to pellet the debris. An aliquot of 50 µg of protein extract was transferred to CPRG solution (1 mM chlorophenol red b-D-galactopyranoside in assay buffer) and the O.D. at 574 nm was measured at intervals over a 2-hour period. The β-galactosidase activity was calculated as a function of the change in O.D.

## Results

### The ADD domain of Atrx is highly conserved in *Drosophila* and other invertebrates

We searched for genes encoding proteins with the ADD domain in the *Drosophila* genome using the ADD region (aa 169-268) of the hATRX protein with the BLAST (Basic Local Alignment Search Tool) of the NCBI (National Center of Biotechnology Information). Using this approach, we identified the *CG8290* annotated gene, which putatively encodes three annotated proteins that have a conserved amino-terminal ADD domain that is 52% homologous and 36% identical to the hATRX ADD domain (Fig. 1A, B). Protein alignment (Fig. 1A) showed that there is a high conservation at the position of the cysteins that coordinate de zinc atoms in the GATA-like and PHD zinc fingers. This conservation is not as high when we align DNMT3L, a methyltransferase that also has an ADD domain (data not shown). The ADD domain in the *Drosophila CG8290* encoded proteins is more similar to the one in hATRX than in DNMT3L (data not shown). While this work was in preparation for publication Alekseyenko *et al*., (2014) named the *CG8290* gene *dadd1* [11].

In the hATRX protein, there are some amino acids in a "hydrophobic pocket" that mediate the interaction between the H3K9me3 and unmethylated H3K4 (H3K4me0) histone tail combination. In general, it is noteworthy that the whole "hydrophobic pocket" binding site is conserved between the ADD in *Drosophila* and in hATRX (aa 110-126 in dAdd1) [33]. There are also many conserved amino acids that are not part of the "hydrophobic pocket", such as the histidine 189 and proline 190 of hATRX (which correspond to aa 96 and 97 in dAdd1) that are mutated in ATRX syndrome [22]. We constructed a three-dimensional model of the ADD domain found in the dAdd1 proteins (aa 64 to 164) and compared it to the hATRX ADD domain (Fig. 1B). The human and *Drosophila* domains mainly overlap in the GATA-like and PHD zinc fingers helices 1, 2 and 3 (h1, h2 and h3, respectively), and the pocket that is important for recognition of H3K9me3 and H3K4me0 is also conserved (Fig. 1C). In the recently published work by Alekseyenko *et al*., (2014), the capability of this domain to bind to the H3K9me3 tail was assayed, confirming it preferentially binds to this histone modification [11].

We also compared the native structure of the ADD domain of dAdd1 to the ADD domain of DNMT3L (data not shown) and found less overlaping when we superimpose the structures. This led us to propose that during ATRX evolution, the protein may have undergone a fission event. To obtain insight into the evolutionary history of ATRX, we decided to perform a rooted phylogenetic inference analysis using homologous sequences of the ADD and the helicase/ATPase domains from ATRX of higher eukaryotes. We found that the shared common ancestor of higher eukaryotes possessed a protein with both the ADD and helicase/ATPase domains, but in insects, it underwent a fission event by which the two domains were separated, generating two different genes (Fig. 1D, Fig. S2) (perhaps involving gene duplication with subsequent partial degeneration, as has been proposed for cmi and TRR proteins or the *monkey king* (*mkg*) gene family in *Drosophila*) [34], [35].

Another interesting feature is that, in *Drosophila,* ADD harboring-proteins acquired other domains such as MADF (myb/Sant-like domain in Adf1, shown in blue hexagons), suggesting novel functions for these proteins (Fig. 1D). MADF domains can recognize repetitive sequences on DNA, for instance in Adf1 and Dip3 the MADF domain directs the binding of these transcription factors to specific promoter sequences [36]. All these data led us to propose that the proteins encoded by *dadd1* could represent the orthologs of the amino terminal half of the hATRX protein.

### The dAdd1 proteins are expressed throughout development and are preferentially nuclear proteins that can bind to different chromatin regions

**Figure 2:**
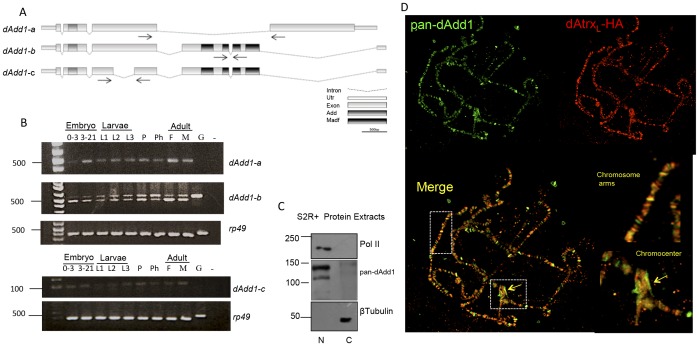
The dAdd1 proteins are expressed throughout development. A. Scheme of the *dadd1* mRNAs generated by alternative splicing. The nucleotide sequence of the ADD and MADF domains are represented as gray boxes. Primers used to amplify cDNAs representing mRNAs encoding Add isoforms are indicated by horizontal arrows. B. The *dadd1* transcripts were detected throughout all *Drosophila* stages of development by RT-PCR. Detection of exons from different *dadd1* mRNAs in cDNA generated from total RNA isolated from embryos (0–3 and 3–21 hour), 1^st^, 2^nd^ and 3^rd^ instar larvae (L1, L2 and L3), pupae (P), pharate (Ph) and female and male adults (F and M). *rp49* was used as a RT- PCR control. Sequence of the specific primers used to detect and sequence exons from each different *dadd1* mRNA are described in Material and Methods section. Molecular weight markers on the left of the panels represent base pairs. C. The dAdd1 (a-c) protein isoforms are mainly nuclear. Detection of dAdd1 (a-c) proteins by Western blotting using the pan-dAdd1 antibody in nuclear (N) and cytoplasmic (C) soluble fractions isolated from S2R^+^ cells. The largest RNA polymerase II subunit and β-tubulin were used as controls of nuclear and cytoplasmic fractions, respectively. Molecular weight markers on the left of the panels represent kDa. D. dAdd1 proteins co-localize with dAtrx protein in polytene chromosomes of third instar larvae. dAtrx_L_-HA and dAdd1 were followed with an anti-HA (red, right upper panel) and the pan-Add1 (green, left upper panel) antibodies respectively. The dAdd1 and dAtrx_L_-HA proteins colocalize in some bands and interbands (left lower panel). They also co-localize in heterochromatic regions such as the chromocenter (see Insets, lower panel right). Note that not all the bands co-localize.

Through RT-PCR analyses of cDNAs obtained from different developmental stages (see Material and Methods and Fig. 2A), we found three transcripts *dAdd1-a*, *dAdd1-b* and *dAdd1-c* (Fig. 2A, and B top panel). The transcripts (*dAdd1-a*, *dAdd1-b*, *dAdd1-c*) corresponded to the ones derived from alternatively spliced transcripts of *CG8290* described in FlyBase [37]. All of these transcripts are deposited maternally into the embryo and are later expressed through all stages of development (Fig. 2B, 0-3 lanes).

Interestingly when we amplify the *dAdd1-b* transcript, another higher molecular weight band appears (Fig. 2B top panel). We sequenced this fragment and it corresponds to a transcript that retains the fifth intron. The presence of this putative transcript varies throughout development being more abundant in larval stages. Bioinformatic analyses indicate that retention of the fifth intron in this transcript would generate a stop codon which will in turn translate into a protein that conserves only one MADF domain. Further analyses are being carried out to assess the presence of this putative protein.

We found that there are three proteins derived from these transcripts that have similar predicted molecular weights ranging from 112 kDa to 130 kDa (Fig. 1C). Interestingly, the dAdd1-b and dAdd1-c proteins have several MADF domains in their carboxy-termini. We designed an antibody that recognizes the three dAdd1 isoforms (called pan-dAdd1, see Materials and Methods), and we tested the antibody's specificity through Western blot and depletion with a GST-dAdd1 fusion protein (Fig. S1A and Materials and Methods section). In the immunoblots we observe two main bands (112 and 130 kDa). Based on the predicted molecular weight of the proteins, the signals of these high-molecular weight bands probably represent the three proteins but cannot be resolved by standard SDS-PAGE electrophoresis.

We found that the dAdd1 proteins are enriched in the nuclear fraction of S2R^+^ cells (Fig. 2C). Because the proteins are nuclear and have putative chromatin and DNA binding domains, we analyzed their location on chromatin. We made polytene chromosomes preparations from third instar larvae, and using immunostaining with the pan-dAdd1 antibody, we found that the dAdd1 proteins are located in several bands and interbands in the polytene arms (Fig. S3). The signal is also present in heterochromatic regions such as the chromocenter and on the fourth chromosome, and it co-localizes with the dAtrx signal in this region and in other heterochromatic regions (Fig. 2D and Fig. S3).

To determine whether dAdd1 colocalizes with dAtrx, we expressed an epitope tagged-dAtrx_L_ (dAtrx_L_-HA) [10] in larval salivary glands. This tagged dAtrx_L_-HA version localizes to the same sites as wild type dAtrx_L_ [25] and does not alter dAdd1 distribution (compare Fig. 2D and Fig. S3). We found that dAdd1 colocalizes with dAtrx_L_-HA in the chromocenter and in other heterochromatic regions (Fig. 2D insets).

These results opened the possibility that dAdd1 proteins with an ADD domain could interact with the ADD-less *Drosophila* dAtrx and cooperate in some cellular functions in the organism.

### The dAdd1 proteins interact directly with the dAtrx_L_ protein

**Figure 3:**
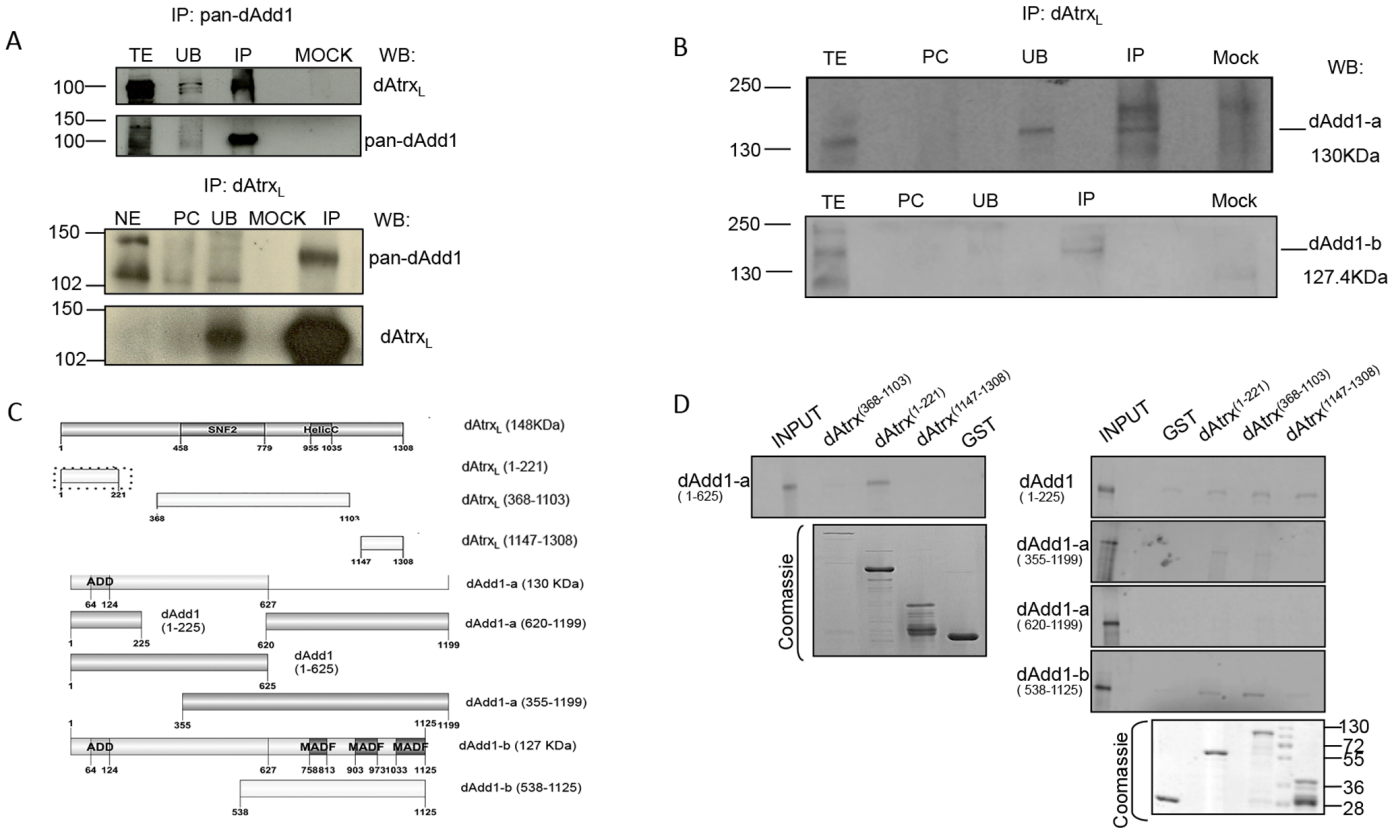
The dAtrx and dAdd1 proteins physically interact. A. Co-immunoprecipitation (CoIP) assays of dAdd1 and dAtrx_L_. IP performed with pan-dAdd1 antibody (top panels) and the reciprocal CoIP assay with anti-dAtrx_L_ antibody (bottom panels). Embryo nuclear extract (NE); S2R^+^ cells total extract (TE); pre-clearing (PC); unbound protein fraction (UB); Mock is the IP performed with an unrelated antibody. The presence of dAdd1 and dAtrx_L_ in the precipitated proteins was determined by Western blotting using the specific antibody. 10% of the nuclear extract (corresponding to approximately 50 µg of protein) used for the IP was loaded as the INPUT fraction. 20% of the total extract (corresponding to 150 µg of protein) was loaded as the INPUT fraction. Molecular weight markers on the left of the panels represent kDa. B. CoIP assay of dAdd1-a and dAdd1-b with dAtrx_L._ S2R+ cells total extract (TE); pre-clearing (PC); unbound protein fraction (UB); IP was performed with anti-dAtrx_L_. Mock is the IP performed with an unrelated antibody. The presence of dAdd1-a and dAdd1-b in the precipitated proteins was determined using the specific antibody. 20% of the total extract (corresponding to 150 µg of protein) was loaded as the INPUT fraction. The 250 kDa band observed in the INPUT lane is an unspecific band. Molecular weight markers on the left of the panels represent kDa. C. Representation of the fragments assayed by Pull-down. The important fragments for the interaction between dAtrx and dAdd are shown in dashed boxes. D. Pull-down assay. The first lane of all panels (except the coomassie panels) shows the 10% of the total amount of transcription/translation labeled protein (dAdd1 fragments) used for each experiment (input). The rest of the lanes are the experimental interaction (GST-dAtrx fragments and dAdd1 fragments) and the control interaction (GST and dAdd fragments) for each analyzed polypeptide. Coomasie staining of the loaded GST-fused proteins is shown in the bottom panels.

To test the proposed interaction hypothesis, we performed co-immunoprecipitation experiments using different antibodies generated against the dAdd1 proteins and dAtrx_L_ (see Materials and Methods). As a first approach we coimmunoprecipitated dAdd1 with the pan-dAdd1 antibody and tested whether we could detect dAtrx_L_. In addition to detecting dAdd1 (Fig. 3A, top panel, pan-dAdd1 blot) we found dAtrx_L_ in the immunoprecipitate (Fig. 3A top panel, dAtrx_L_ blot, IP lane), the specific bands obtained in the IP are marked with a white arrow. We also performed the reciprocal immunoprecipitation with the dAtrx_L_ antibody, and we show that at least one of the dAdd1 proteins, approximately 130 kDa, co-immunoprecipitates with dAtrx_L_ (Fig. 3A bottom panel, IP lane) the specific band is marked with a black arrow. The signal in both cases is specific because it cannot be detected when we used an irrelevant antibody to perform the immunoprecipitation assay (mock lane in all the blots).

To further characterize which of the dAdd1 proteins are in the observed immunoprecipitated band, we performed a coimmunoprecipitation assay using S2R^+^ total extracts with antibodies generated against dAdd1-a or dAdd1-b. We found that both, dAdd1-a and dAdd1-b coimmunoprecipitate with dAtrx_L_ (Fig. 3B, top and bottom panels), the white arrows indicate the specific bands corresponding to the co-immunoprecipitated proteins. In the dAdd1-a blot (Fig. 3B, top panel) there is a high molecular weight band, near the 250 kDa weight marker, this band is unspecific because it also appears in the immunoprecipitation performed with a purified rabbit IgG antibody (Fig. 3B, mock lane). The fact that both proteins, dAdd1-a and dAdd1-b, can immunoprecipitate with dAtrx_L_ indicates that the interaction domain is most likely conserved in both proteins or that a third protein is mediating the interaction.

To map the dAdd1 protein domain(s) important for the interaction with dAtrx_L_, we performed a series of pull-down assays. We generated several fragments of dAtrx_L_ and dAdd1 proteins and expressed them as GST-fusion proteins in a bacterial system or expressed and labeled them with S^35^ using an *in vitro* transcription/translation system (see Materials and Methods, Fig. 3C). We tested four different fragments of the dAdd1-a protein. Two of these fragments are present in the three isoforms (dAdd1 1-225, dAdd1 1-625). When we assayed the dAdd1 1-225 fragment with three different dAtrx_L_ fragments, we observed that it binds to all of the tested fragments of dAtrx_L,_ but this fragment also binds to the negative control GST, although with less affinity (Fig. 3D top panel on the right). In contrast, the larger fragment that contains aminoacids 226-625 (fragment dAdd1 1-625) binds specifically to the amino-terminal fragment of dAtrx_L_ (dAtrx 1-221) (Fig. 3D top left panel).

We also tested the dAdd1-a (355-1199) and dAdd1-a (620-1199) fragments. The first fragment, which contains amino acids 355-625 binds to two dAtrx_L_ fragments (1-221 and 368-1103), corresponding to the amino-terminal and SNF2 fragments, whereas the second dAdd1-a fragment (620-1199), which lacks amino acids 355-625, fails to interact with any of the tested dAtrx_L_ fragments (compare second and third panels from top to bottom on the right of Fig. 3D).

In these analyses, we also found that a fragment that includes the MADF domains (dAdd1-b 538-1125) interacts with the amino terminal and SNF2-containing fragments of the dAtrx_L_ protein (Fig. 3D from top to bottom: fourth panel on the right) but this fragment also binds to the negative control GST.

From these data, we conclude that the dAdd1 domain that interacts with dAtrx_L_ is conserved in all dAdd1 isoforms. Thus, all dAdd1 isoforms can directly interact with the amino-terminal domain of dAtrx_L_ (a.a. 1-221) through their amino-terminal domain (a.a. 1-625). Interestingly, this dAtrx_L_ fragment is only conserved in the long isoform, and it does not overlap with the HP1a interaction domain [22]. In contrast, it does overlap with the DREF interaction domain, which was reported by our group [25]. Based on these results, we conclude all dAdd1 isoforms can interact directly with the dAtrx_L_ protein.

### *atrx* and *dadd1* interact genetically

At this point we have demonstrated that dAdd1 and dAtrx physically interact; and therefore, we wanted to know whether they genetically interact in the fly. For this purpose, we made transheterozygous *atrx* and *dadd1* flies. For these analyses, we used three *atrx* alleles [20]. *atrx^1^* is a deficiency that uncovers dAtrx as well as three more adjacent genes; *atrx^2^* is an hypomorphic allele that affects both the long and short dAtrx isoforms and *atrx^3^* is an hypomorphic allele that affects only the long isoform. The survival of heteroallelic *atrx* flies is affected, as previously reported, and they do not present any other obvious phenotype (Table 1, [10]).

**Table 1:** Interaction between *dadd1*, and *atrx*.

Genotype	Viability^a^ (%)	Melanotic Masses^b^ (%)
*atrx^1^*/+	789/789 (100)	0/789 (0)
*atrx^2^*/+	202/202 (100)	0/202 (0)
*atrx^3^*/+	775/789 (98)	0/775 (0)
*atrx^1^*/*atrx^2^*	80/202 (40)	0/80 (0)
*atrx^1^*/*atrx^3^*	387/789 (49)	0/387 (0)
*atrx^2^*/*atrx^3^*	130/228 (57)	0/130 (0)
*dadd1^NP1240^*/+	180/180 (100)	0/180 (0)
*dadd1^NP0793^*/+	255/255 (100)	0/255 (0)
*dadd1^NP1240^*/*dadd1^NP1240^*	91/206 (44)	0/91 (0)
*dadd1^NP1240^*/*dadd1^NP0793b^*	163/180 (90)	0/163 (0)
*dadd1^NP1240^*/+;*atrx^1^*/+	454/454 (100)	32/454 (7)
*dadd1^NP1240^*/+;*atrx^2^*/+	568/568 (100)	11/568 (2)
*dadd1^NP1240^*/+;*atrx^3^*/+	632/632 (100)	14/632 (2)
*dadd1^NP0793^*/+;*atrx^1^*/+	152/152 (100)	5/152 (3)
*dadd1^NP0793^*/+;*atrx^2^*/+	322/322 (100)	11/322 (3)
*dadd1^NP0793^*/+;*atrx^3^*/+	243/243 (100)	7/243 (3)
*dadd1^NP1240^*/+;*atrx^1^*/*atrx^3^*	76/105 (72)	9/76 (12)
*dadd1^NP1240^*/+;*atrx^2^*/*atrx^3^*	106/141 (75)	8/106 (8)
*dadd1^NP0793^*/+;*atrx^2^*/*atrx^3^*	161/219 (73)	15/161 (9)

a The number of flies obtained over the number of flies expected according to the healthiest class in each cross. Percentage is in parentheses.

b Number of adult individuals with melanotic masses (Fig. 4B) observed over the total number of that particular class. Percentage is in parentheses.

We also used two *P*-element insertion lines, *dadd1^NP1240^* and d*add1^NP0793^.* These insertions lie at 83 or 81 bp, respectively, from the *dadd1* transcriptional start site (Fig. 4A). To determine whether the insertions affected the levels of the dAdd1 proteins, we extracted total proteins from adult flies of the different genotypes and analyzed the presence of the dAdd1 proteins through Western blotting using the pan-dAdd1 antibody (Fig. 4B). We found that the levels of the dAdd1 proteins are diminished in homozygous *dadd1^NP1240^*/*dadd1^NP1240^* individuals, with respect to the heterozygous *dadd1^NP1240^/+* or +/+ flies (Fig. 4B). We also analyzed the *dadd1* mRNA levels of *dadd1^NP1240^/+* or *dadd1^NP0793^/+* individuals through semi-quantitative RT-PCR and found that the *dadd1* mRNA levels are diminished in the mutant individuals compared to the *dadd1* wild type flies (Fig. S4). These results indicate that these alleles are hypomorphic. Similar results were obtained with the *dadd1^NP1240^*/*dadd1^NP0793^* heteroallelic flies (data not shown) and thus we used for the rest of our tests the *dadd1^NP1240^* allele. 44% and 90% of the *dadd1^NP1240^/dadd1^NP1240^* and *dadd1^NP1240^/dadd1^NP0793^* flies reach adulthood respectively, showing that the higher lethality present in *dadd1^NP1240^/dadd1^NP1240^* individuals (56%) compared to the one presented by the *dadd1^NP1240^/dadd1^NP0793^* flies (only 10%) may be caused by other lethals present in the *dadd1^NP1240^* chromosome (Table 1). The heteroallelic *dadd1^NP1240^*/*dadd1^NP0793^* individuals are fertile and do not present any other obvious phenotype.

**Figure 4:**
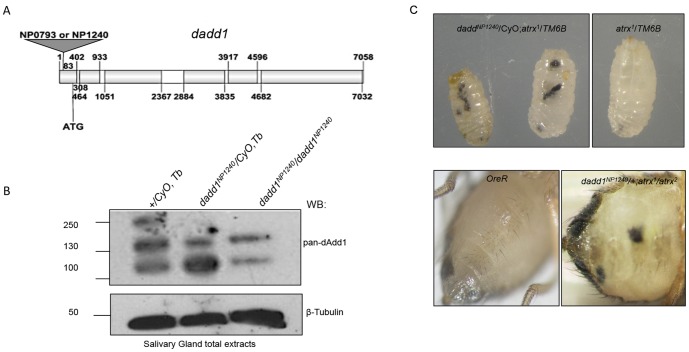
Genetic interaction between *dadd1* and *atrx*. A. Scheme of *dadd1* showing the position of the *EP* elements insertions (gray triangle) in *dadd1^NP1240^* and *dadd1^NP0793^* alleles, introns are represented by white boxes while exons are represented by gray boxes. The start codon at position 308 pb is also shown. *B. dadd1^NP1240^* is an hypomophic *dadd1* allele. Western blotting assay probed with the pan-dAdd1 antibody. Each lane contains 150 µg of proteins from adult flies of the indicated genotype. β-tubulin was used as a loading control. Note the low levels of the dAdd1 proteins in homozygous individuals with respect to +/+ individuals. This demonstrates the allele is hypomorphic. The 250 kDa band observed in the wild type lane is an unspecific band. Molecular weight markers on the left of the panels represent kDa. C. Some *dadd1*/*atrx* individuals present melanotic masses. Melanotic masses that appear during larval (upper panel) and adult stages (lower panel) of mutant *dadd1/atrx* individuals are shown. Wild type individuals (*w^1118^* for larvae and *OreR* for adult) are shown to the left for comparison.

Survival of the transheterozygous *dadd1/atrx* flies is mildly affected, and a small percentage of these flies present melanotic masses, both in third instar larvae and adult individuals. The percentage of melanotic masses is higher in flies carrying two different alleles of *atrx* in combination with one allele of *dadd1* (Fig. 4, Table 1). We obtained similar results for all the combinations of *atrx* and *dadd1* alleles (Fig. 4, Table 1,). The masses also appear during larval stages, and these individuals fail to advance further in development (Fig. 4C). Similarly, adult flies presenting melanotic tumors die within the first 10 days post-eclosion (data not shown). These data provide evidence that, besides the physical interaction we showed in the previous sections, there is a genetic interaction between dAdd1 and Atrx functions that is essential for fly development.

### The dAdd1 proteins co-localize with HP1a in heterochromatic regions and cooperate with dAtrx in the maintenance of pericentric heterochromatin

As dAdd1 proteins interact with dAtrx_L_, we thought that they could be involved in heterochromatin maintenance; therefore, we analyzed their interaction with HP1a.

**Figure 5:**
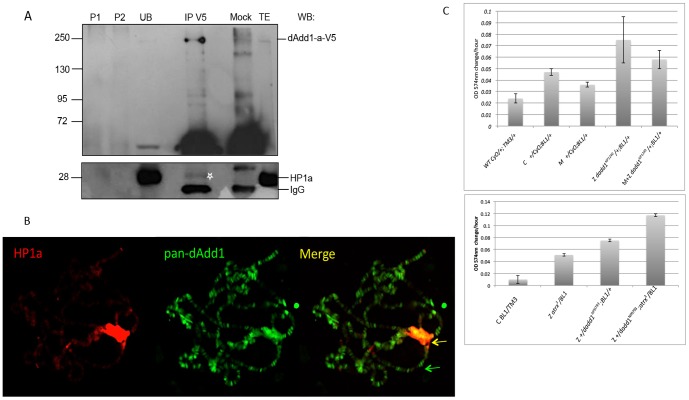
The dAdd1 proteins co-localize in heterochromatic regions with HP1a and cooperate with dAtrx in the maintenance of pericentric heterochromatin. A. CoIP assay of dAdd1-a and HP1a. Total extract (TE) from transiently transfected S2R^+^ cells with dAdd1-a-V5, P1 and P2 are pre-clearing 1 and 2 (see Material and Methods); unbound protein fraction (UB); IP performed with anti-V5 (upper panel); Mock is the IP performed with purified mouse IgG. The presence of dAdd1-a-V5 was determined using the anti-V5 antibody. The presence of HP1a was determined using the anti-HP1a (CIA9) antibody (lower panel). Molecular weight markers on the left of the panels represent kDa. B. Polytene chromosomes preparations from wild-type *OreR* third instar larvae were simultaneously stained with anti-HP1a (red signal) and pan-dAdd1 (a-c) antibody (green signal). Merge bands containing both factors can be visualized mainly in the chromocenter (yellow signal). *C. dadd1* is a suppressor of position effect variegation. Quantitative β-galactosidase assay. The graphs show that *dadd1* alleles can suppress position effect variegation measured by the enzymatic activity of Lac-Z. WT =  wild type flies with *CyO* and *TM3* chromosome balancers; C =  control flies derived from F1 progeny of paternally delivered *dadd1* alleles; M =  Maternal depletion, F1 progeny flies of maternally delivered *dadd1* alleles; Z = zygotic depletion, flies derived from crossing males carrying the *dadd1* alleles to females carrying the *BL1* allele; M+Z =  maternal and zygotic depletion, flies derived from crossing females carrying the *dadd1* alleles to males carrying the *BL1* reporter allele. The bottom graph shows that transheterozygous *dadd1, atrx* flies have an additive effect in the suppression of position effect variegation.

As mentioned before, the HP1α interaction domain in the dAtrx_L_ protein (245aa-CxVxL-249aa, [10]) does not overlap with the interaction domains of the dAdd1 proteins. It is possible that dAdd1-a, b or c, dAtrx_L_ and HP1a can co-exist in the same protein complex. If this is true, then we should be able to coimmunoprecipitate HP1α with the dAdd1 proteins. We expressed a V5 epitope-tagged dAdd1 version in S2R^+^ cells and detected the presence of HP1a in an immunoprecipitation (IP) assay of the V5-dAdd1 protein with the V5 antibody (Fig. 5A, lower panel, white asterisk). Although the signal is faint it is specific because it cannot be detected in the mock control IP (Fig. 5A). This finding indicates that dAdd1-a is able to co-immunoprecipitate with HP1a.

We also show that dAdd1 and HP1a co-localize in heterochromatin regions and in the chromocenter of wild type polytene chromosomes (Fig. 5B yellow arrow). The dAdd1 signal is also observed in regions where no HP1a signal is detected (Fig. 5B green arrow).

The coimmunoprecipitation and immunolocalization analyses suggest that the dAdd1 proteins could be located in different regions of the chromatin. To analyze the binding of these factors at several heterochromatic and euchromatic regions in more detail, we performed chromatin immunoprecipitation (ChIP) assays using different antibodies against HP1a, dAtrx_L_ and the dAdd1 proteins with chromatin from third instar larvae salivary glands and S2R^+^ cells (see Material and Methods).

HP1α is not enriched in the TAS (Telomeric Associated Sequences) regions [38]. On the other hand, Antão *et al*., (2012) [30] found through a proteomic analysis of chromatin segments (PICh) that dAtrx and of the product of *CG8290* are enriched in the telomeric associated sequences (TAS-L) but they did not find HP1a. Thus, we looked for the presence of HP1α, dAtrx_L_ and the dAdd1 proteins in the telomeric region (TAS-L). Our ChIP experiments show, similar to Antão *et al*., (2012) [30], that dAtrx_L_ and the proteins, but not HP1α, are enriched in the TAS-L region (Fig. S5). We also found that dAtrx_L_ and the dAdd1 proteins colocalize in a region that can potentially form a G quadruplex structure (G4) (Fig. S5).

In search for heterochromatic regions where the proteins did not co-localize we tested the rover retrotransposon, which we know is a constitutive heterochromatic region enriched in H3K9me3 mark [29]. Here we found dAtrx_L_ is present but the dAdd1 proteins are not enriched in this region. This result indicates that dAdd1 and dAtrx_L_ do not colocalize at all the heterochromatic regions in the genome (Fig. S5). Next, we looked for an euchromatic region, we decided to use the *sgs8* promoter which is actively transcribing in third instar salivary glands [29]. We observed that in this euchromatic region there is some enrichment of dAdd1 proteins whereas dAtrx_L_ is not enriched as expected (Fig. S5). This finding is supported by the fact that in the immunolocalization experiments shown above, we also observe dAdd1 in euchromatic regions in the chromosome arms (Fig. 2D and Fig. S3).

The results obtained in the ChIP analyses could represent the capability of the different dAdd1 proteins to bind different regions of the chromatin. The fact that dAtrx_L_ and the dAdd1 proteins are not always together, as shown in the ChIP and the immunolocalization analyses, provide evidence that the dAdd1 proteins have functions independent of that with dAtrx_L_. This result also shows that dAtrx_L_ can bind to chromatin regions independent of the dAdd1 proteins, most likely through interactions with other factors such as DREF [23].

The colocalization of HP1a, dAtrx_L_ and dAdd1 in the chromocenter raises the possibility that the dAdd1 proteins are involved in the maintenance of heterochromatin. In a model for the maintenance of pericentric heterochromatin proposed by Eustermann S, *et al*., (2011) [4], the ADD domain of the hATRX protein, reinforced by the interaction with HP1a, recognizes pericentric heterochromatin. Once hATRX is recruited its helicase/ATPAse domain directs deposition of the histone variant H3.3 [4].

It has not been determined conclusively whether Drosophila dAtrx is involved in the deposition of the histone variant H3.3, but both HP1a and dAtrx_L_ localize at the chromocenter of polytene chromosomes and act as suppressors of position effect variegation for the *w^m4^* allele, an inversion that lies near pericentric heterochromatin [10].

Following this hypothesis we tested the *dadd1* capability to suppress position effect variegation. We used the BL1 line, which carries a LacZ reporter construct that lies near the centromere [24], [32]. In this assay, we can determine whether *dadd1* is involved in the establishment, crossing virgin females carrying the *dadd1* alleles to males carrying the reporter BL1 (maternal effect), or maintenance, crossing males carrying the *dadd1* alleles to females carrying the reporter BL1 (zygotic effect), of heterochromatin [32] (see Material and Methods).

The results show that *dadd1* does not have a relevant role in the establishment of pericentric heterochromatin (compare M (maternal) bar and C (control) bar in Fig. 5C top graph). When we analyzed the zygotic effect, virgin BL1 females crossed to *dadd1* male carrying alleles, the activity of LacZ was consistently higher in *dadd1* mutants (compare Z (zygotic) bar to C (control) bar in Fig. 5C top graph), indicating that the *dadd1* alleles can suppress position effect variegation and that *dadd1* has a role in the maintenance of heterochromatin rather than in the establishment of heterochromatin.

Next, we tested whether a combination of *dadd1* and *atrx* alleles enhances the suppression of position effect variegation in a cooperative manner. In fact, this is what we observed. Our results show that transheterozygous *dadd1*/*atrx* flies suppress position effect variegation more efficiently than heteroallelic flies carrying either the *dadd1* or *atrx* alleles (compare third and fourth bars in Fig. 5C bottom graph).

Overall, these results indicate that dAdd1 proteins are capable of interacting with proteins, such as HP1a, that are involved in the establishment of heterochromatin and that they cooperate with dAtrx_L_ in the maintenance of heterochromatin.

### *Su(var)205* interacts genetically with *dadd1*, and *atrx*

We showed that *dadd1*/*atrx* individuals present melanotic masses. The *Su(var)205^2^* is a loss-of-function HP1a allele because it carries a mutation in aminoacid 26 within the chromodomain. We tested whether the loss of function of HP1a in the presence of *dadd1* and/or *atrx* mutations affects the presence of the melanotic masses found in *dadd1/atrx* individuals.

Transheterozygous *Su(var)205^2^* flies carrying the *dadd1*, or *atrx^1^* or *atrx^2^* alleles do not present melanotic masses, whereas *Su(var)205^2^* flies carrying *atrx^3^* (which affects only the long isoform of dAtrx) present melanotic masses in a small percentage (Table 2). The penetrance of melanotic masses in *dadd^NP1240^*/+; *atrx^3^*/+ (4%) or *Su(var)205^2^*/+; *atrx^3^*/+ (2%) individuals increases to 14% in *Su(var)205^2^*/*dadd^NP1240^*;*atrx^3^*/+ individuals where mutations in the three genes are together (Table 2). These data show that the presence of melanotic masses in transheterozygous *dadd1*, *atrx* and *Su(var)205* flies most likely involves misregulation in the maintenance of heterochromatin.

**Table 2:** *Su(var)205* interaction with *dadd1*, and *atrx*.

Genotype	Melanotic Masses a(%)
*Su(var)205^2^*/+	0/487 (0)
*Su(var)205^2^*/+;*atrx^3^*/+	6/366 (2)
*Su(var)205^2^*/*dadd1^NP1240^*	0/201 (0)
*dadd1^NP1240^*/+;*atrx^3^*/+	3/69 (4)
*Su(var)205^2^*/*dadd1^NP1240^*; *atrx^3^*/+	16/111(14)

a Number of adult individuals with melanotic masses (Fig. 4B) observed over the total number of that particular class. Percentage is in parentheses.

## Discussion

In vertebrates, hATRX is a protein with an ADD domain and an SNF2-helicase/ATPase motif. Through evolution, the SNF2 domain of hATRX has been highly conserved, but in invertebrates, the ADD domain is lost (Fig. 1D, [39]). In this article, we describe the characterization of the *Drosophila* dAdd1 proteins as orthologs to the amino-terminal region of Atrx. The *dadd1* gene expresses the dAdd1 proteins, which have an ADD domain (Fig. 1C), throughout development (Fig. 2B). The 3D structure of the dAdd proteins ADD domain (Fig. 1B) is more similar to the one found in hATRX than to the ADD domains found in other proteins. *Drosophila* dAdd1 and the ADD-less dAtrx interact physically (Fig. 3A, 3D) and genetically (Tables 1 and 2), and they interact with HP1a in heterochromatic regions (Fig. 5B).

The human *hATRX* gene encodes an SNF2 helicase/ATPase protein that has many different functions. It is part of a complex that includes the histone variant H3.3 chaperone DAXX, and it is involved in the deposition of this histone variant in pericentric and telomeric heterochromatin [7], [40], [41]. The hATRX protein can also bind to regions in the genome that can potentially form G-quadruplex structures and it has been proposed that this binding alleviates the quadruplex conformation and allows the deposition of H3.3 [9], [42]. Its participation in the deposition of histone variant H3.3 requires the helicase/ATPase activity, although it has not been determined whether this activity is also required for the binding and recognition of the G-quadruplex structures of the DNA. In addition to the SNF2 helicase/ATPase domain, hATRX also has an amino-terminal domain composed of two zinc fingers, a GATA-like finger and a PHD finger called the ADD domain. This domain is able to recognize the H3K9me3 histone mark in combination with unmodified H3K4 through a "hydrophobic pocket", which is an unusual feature for a PHD zinc finger [33]. The interaction between hATRX and histone H3 chromatin marks has been shown to maintain hATRX binding to pericentric heterochromatin. A model has been proposed in which hATRX is directed to pericentric heterochromatin through the ADD domain, and this interaction is reinforced by the interaction between hATRX and HP1a. In this model, the SNF2 helicase/ATPase domain then directs histone H3.3 deposition or performs other required ATP-dependent functions [4].

*dadd1* encodes three polypeptides that contain an ADD domain that is highly similar to the hATRX ADD domain (36% identity and 52% of similarity, [11] and this work). The comparison of the 3D-structure analyses of the ADD of *Drosophila* dAdd1 and hATRX reveal that these domains overlap and that the histone recognition pocket is conserved.

Our phylogenetic inference analysis using the ADD domain of hATRX indicates that the ATRX gene underwent a fission event. This fission has been described in at least another chromatin-binding-protein encoding gene, *cara mitad* (*cmi*), which is the homolog of the amino-terminal portions of mammalian *MLL2* and *MLL3* [32].

It is intriguing that there are three spliced isoforms encoded by the *dadd1* gene. The dAdd1-a isoform contains the ADD domain and no other putative domains, while the other two spliced isoforms have additional domains called MADF (myb/SANT-like domain in Adf1). The MADF domains are a subfamily of the SANT domains. They are DNA-binding domains found in all *Drosophila* species. In the case of Adf1, the first *Drosophila* factor identified to have an homology to the Myb oncoprotein [43], the MADF domain recognizes and binds certain sequences in repetitive regions [44], although there are some examples in which the MADF domains can also bind proteins, and apparently the specificity lies in the domain's isoelectric point [45].

The predicted isoelectrical point of the three MADF domains in the dAdd1 proteins is basic, which could indicate that they can bind to DNA.

The acquisition of new domains indicates that these proteins most likely have diverged functions. One indication of this could be the pattern of the immunolocalization of dAdd1 in polytene chromosomes, where not all the signals derived from the pan-dAdd1 antibody co-localize with dAtrx_L_. This indicates that the dAdd1 isoforms could also have roles independent of their interaction with dAtrx_L._

The survival of transheterozygous individuals carrying the *dadd1/atrx* alleles is compromised, and some of the larvae and adult individuals show melanotic masses. We found that these proteins also colocalize with HP1a and that dAdd1-a immunoprecipitates with HP1a. Diminished HP1a levels in *dadd1/atrx* flies enhance the incidence of melanotic masses. Our results are supported by the recent report of Alekseyenko *et al*., (2014), in which it is demonstrated that dAdd1 physically interacts with HP1a and that dAdd1 is a suppressor of variegation [11].

The generation of melanotic masses involves problems in the differentiation of hematopoietic cells in the flies. The differentiation of hematopoietic cells takes place in the early embryonic head mesoderm and in the lymph gland of late larvae, and three different types of cells are derived from the prohemocytes; the plasmatocytes, crystal cells and lamellocytes. Several different pathways regulate the differentiation of these cells [46]. One of the pathways involves a balance in the expression of the Pnr-α and Pnr-β proteins, which is controlled by the JAK/STAT pathway [48]. Jak hyperactivation results in the proliferation of hemocytes, lamellocyte differentiation and the generation of melanized pseudotumors. Pseudotumors or melanotic masses are formed by crystal cells and lamellocytes [47]. On the other hand, Stat is a positive regulator of plasmatocyte differentiation, and one of the downstream factors regulated by Stat is the GATA factor Pannier [48]. Importantly, our group had already identified *pannier (pnr*) as a gene regulated by dAtrx_L_ and another transcriptional factor DREF [25]. In that report, we concluded that dAtrx_L_ is recruited to the *pnr* gene promoter through DREF, and that it acts as a co-repressor of *pnr* gene expression.

Furthermore, the data obtained from the genetic interactions between the *dadd1, atrx* and *Su(var)205^2^* alleles indicates that melanotic masses are derived from problems in the proteins involved in heterochromatin maintenance, such as dAtrx_L_ and HP1a. The position effect variegation assay indicated that the dAdd1 proteins are involved in the maintenance of heterochromatin. Thus, an attractive hypothesis is that dAdd1, dAtrx_L_ and HP1a cooperate and promote heterochromatinization at the promoters of the genes involved in the JAK/STAT pathway, including *pnr*. Lack of these proteins could lead to misregulation of the effectors of the pathway, giving rise to the melanotic masses. Experiments are being carried out to test this hypothesis.

In the human ATRX syndrome, the majority of the mutations identified so far affect the ADD or the SNF2 helicase/ATPAse domains. The fact that the domains are separated in flies provides a new important tool to study the individual roles these domains have in the development of the organism.

## Supporting Information

Figure S1 **dAdd1 antibodies recognize specifically the dAdd1 proteins.** a) Western blot using the pan-dAdd1 antibody. The dAdd1 isoform signals (lane 1) observed with the pan-dAdd1 antibody are no longer observed in lane 2. This demonstrates that the GST-dAdd1 fusion protein which harbors the peptide used to raise the pan-dAdd1 antibody is able to deplete them from this fraction. The dAdd1 signals are no longer observed (lane 2) showing that the pand-dAdd1 specifically recognizes the dAdd1 isoforms (see also Materials and Methods). b) Anti-dAdd1-a antibody recognizes the dAdd1-a protein. Indicated GST fusion proteins were loaded and blotted onto a nitrocelulose membrane. The Western blot was performed with an anti-dAdd1-a antibody (top panel) or an anti-GST antibody (bottom panel). The dAdd1 specific signal is observed only where the GST-dAdda-1 fusion protein harboring the peptide used to raise the antibody was loaded (GST-dAdd1 aminoacids 620-1199) (left lane, top panel). The antibody does not recognize a fusion protein that lacks this peptide (right lane, top panel). The GST antibody recognizes the aforementioned two GST-dAdd1 fusion proteins (bottom panel). Extra bands (asterisks, right lane, bottom panel) may be fusion protein degradation. c) Specificity test for the dAdd1-b antibody. Indicated GST-fusion proteins were induced in *E. coli*. Induced extracts were loaded and blotted onto nitrocellulose membranes. The Western blot (upper panel) was performed using the anti-dAdd1-b antibody. A specific signal is observed (lane 3, black arrow in the blot and in the Ponceau staining) which corresponds to the induced GST-dAdd1-b fragment (aa 530-1125) and not GST or GST-dAdd1-a (aa 620-1199) (lanes 1 and 2 respectively). Faint bands in the first two lanes are unspecific signals.
(TIF)

Figure S2 **The** ***atrx*** **gene suffered a fission event in the Insecta class.** Maximum Likelihood Phylogenetic Analysis of Helicase/ATPase domain and the corresponding Protein Domain Architecture information of its containing proteins. The numbers shown represent bootstrap values. It can be seen that the common ancestor to plants and animals had a protein with both, the ADD and the helicase/ATPase domains, but insects show the homologous domains in different proteins. Since it is more likely that only one fusion event, instead of two independent acquisitions of the same domain, occurred during the evolution, the most parsimonious explanation is to consider a model in which a gene fission event occurred within the Insecta class. For the parameters used, see Material and Methods section.
(DOCX)

Figure S3 **The dAdd1 proteins localize at many chromatin regions in polytene chromosomes.** Wild type polytene chromosome staining was performed with the pan-dAdd1 antibody (red). The dAdd1 proteins localize in heterochromatic regions such as the chromocenter and the fourth chromosome (inset, white arrow). Staining along the chromosomes arms and in euchromatic regions is also observed.
(TIF)

Figure S4 **Alleles** ***dadd1^NP1240^*** **and** ***dadd1^NP0793^*** **are hypomorphs.** Semiquantitative RT-PCR from wild type and mutant *dadd1* flies. dadd1 mRNA level is lower in the *dadd1^NP1240^* (upper panel) and *dadd1^NP0793^* (lower panel) heterozygous flies than in the *dadd1* wild type flies (*w^1118^*). In homozygous *dadd1^NP1240^/dadd1^NP1240^* (upper panel), *dadd1* is even lower that in the heterozygous condition. *rp49* transcript levels remained unchanged in the mutant alleles.
(DOCX)

Figure S5 **The dAdd1 proteins co-localize** ***in vivo*** **with dAtrx_L_ and HP1a in some chromatin regions.** ChIP assay using total extracts from third instar salivary glands (SG) prepared from wild-type larvae and S2R+ cells. Graphs represent the percentage of input precipitated using the different antibodies for the same regions. Note that in the rover region only dAtrx_L_ is enriched. Three independent biological replicates were performed.
(TIF)
