# Correction: Characterization of the *Drosophila* Group Ortholog to the Amino-Terminus of the Alpha-Thalassemia and Mental Retardation X-Linked (ATRX) Vertebrate Protein

**DOI:** 10.1371/journal.pone.0149367

**Published:** 2016-02-10

**Authors:** Brenda López-Falcón, Silvia Meyer-Nava, Benjamín Hernández-Rodríguez, Adam Campos, Daniel Montero, Enrique Rudiño, Martha Vázquez, Mario Zurita, Viviana Valadez-Graham

The following information is missing from the Funding section: Brenda Araceli López Falcón Piza is a recipient of the scholarship registered under number: 176609 of the Consejo Nacional de Ciencia y Tecnología (CONACYT).

Additionally, the following information is missing from the Acknowledgements section: This article was part of the doctoral thesis of the student Brenda Araceli López Falcón Piza who is a student of the PhD: Programa de Doctorado en Ciencias Biomédicas (PDCB) at the Universidad Nacional Autónoma de México. This student receives the scholarship registered under number: 176609 of the Consejo Nacional de Ciencia y Tecnología (CONACYT).
