# Supplementary figures and images for: Characterization of the Drosophila Group Ortholog to the Amino-Terminus of the Alpha-Thalassemia and Mental Retardation X-Linked (ATRX) Vertebrate Protein
Source: PLoS One. 2014 Dec 1;9(12):e113182. doi: 10.1371/journal.pone.0113182 (PMC4249797; doi:10.1371/journal.pone.0113182)

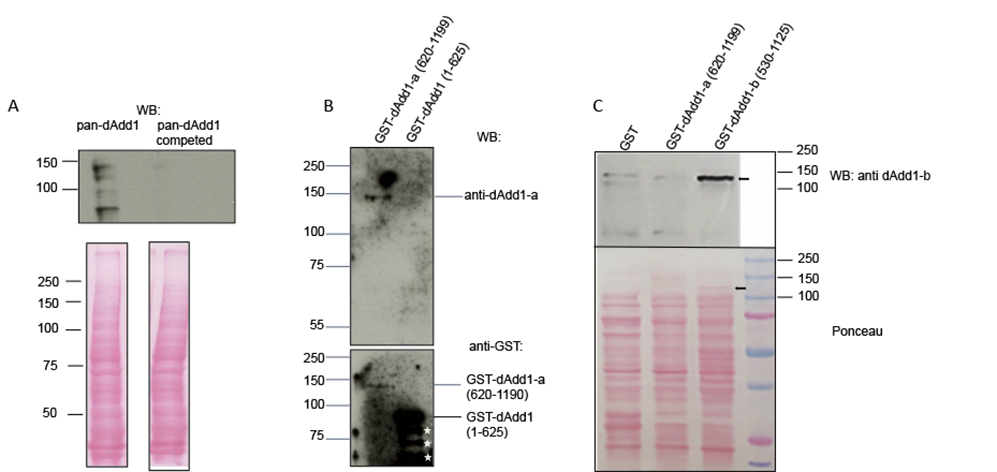

Supplement: Figure S1 — dAdd1 antibodies recognize specifically the dAdd1 proteins. a) Western blot using the pan-dAdd1 antibody. The dAdd1 isoform signals (lane 1) observed with the pan-dAdd1 antibody are no longer observed in lane 2. This demonstrates that the GST-dAdd1 fusion protein which harbors the peptide used to raise the pan-dAdd1 antibody is able to deplete them from this fraction. The dAdd1 signals are no longer observed (lane 2) showing that the pand-dAdd1 specifically recognizes the dAdd1 isoforms (see also Materials and Methods). b) Anti-dAdd1-a antibody recognizes the dAdd1-a protein. Indicated GST fusion proteins were loaded and blotted onto a nitrocelulose membrane. The Western blot was performed with an anti-dAdd1-a antibody (top panel) or an anti-GST antibody (bottom panel). The dAdd1 specific signal is observed only where the GST-dAdda-1 fusion protein harboring the peptide used to raise the antibody was loaded (GST-dAdd1 aminoacids 620-1199) (left lane, top panel). The antibody does not recognize a fusion protein that lacks this peptide (right lane, top panel). The GST antibody recognizes the aforementioned two GST-dAdd1 fusion proteins (bottom panel). Extra bands (asterisks, right lane, bottom panel) may be fusion protein degradation. c) Specificity test for the dAdd1-b antibody. Indicated GST-fusion proteins were induced in E. coli. Induced extracts were loaded and blotted onto nitrocellulose membranes. The Western blot (upper panel) was performed using the anti-dAdd1-b antibody. A specific signal is observed (lane 3, black arrow in the blot and in the Ponceau staining) which corresponds to the induced GST-dAdd1-b fragment (aa 530-1125) and not GST or GST-dAdd1-a (aa 620-1199) (lanes 1 and 2 respectively). Faint bands in the first two lanes are unspecific signals. (TIF) [file pone.0113182.s001.tif]

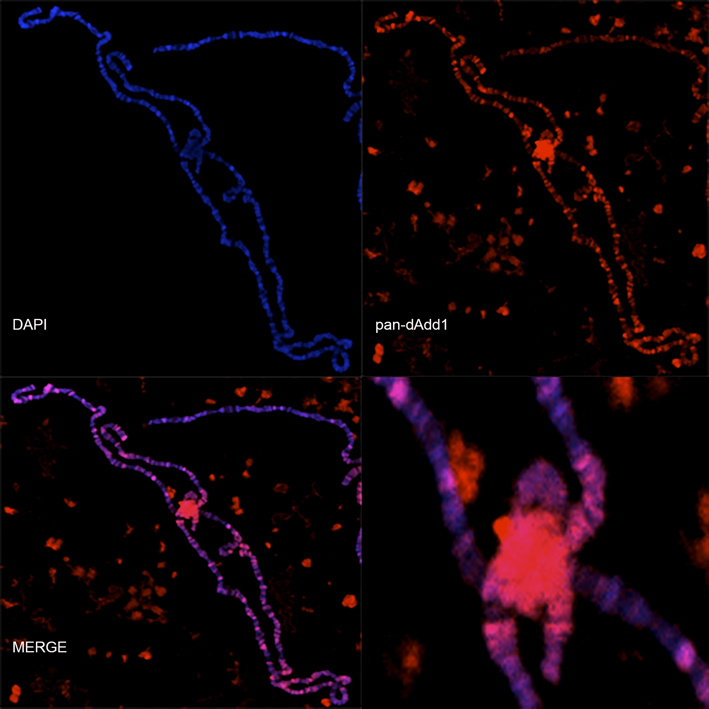

Supplement: Figure S3 — The dAdd1 proteins localize at many chromatin regions in polytene chromosomes. Wild type polytene chromosome staining was performed with the pan-dAdd1 antibody (red). The dAdd1 proteins localize in heterochromatic regions such as the chromocenter and the fourth chromosome (inset, white arrow). Staining along the chromosomes arms and in euchromatic regions is also observed. (TIF) [file pone.0113182.s003.tif]

*dAdd1*


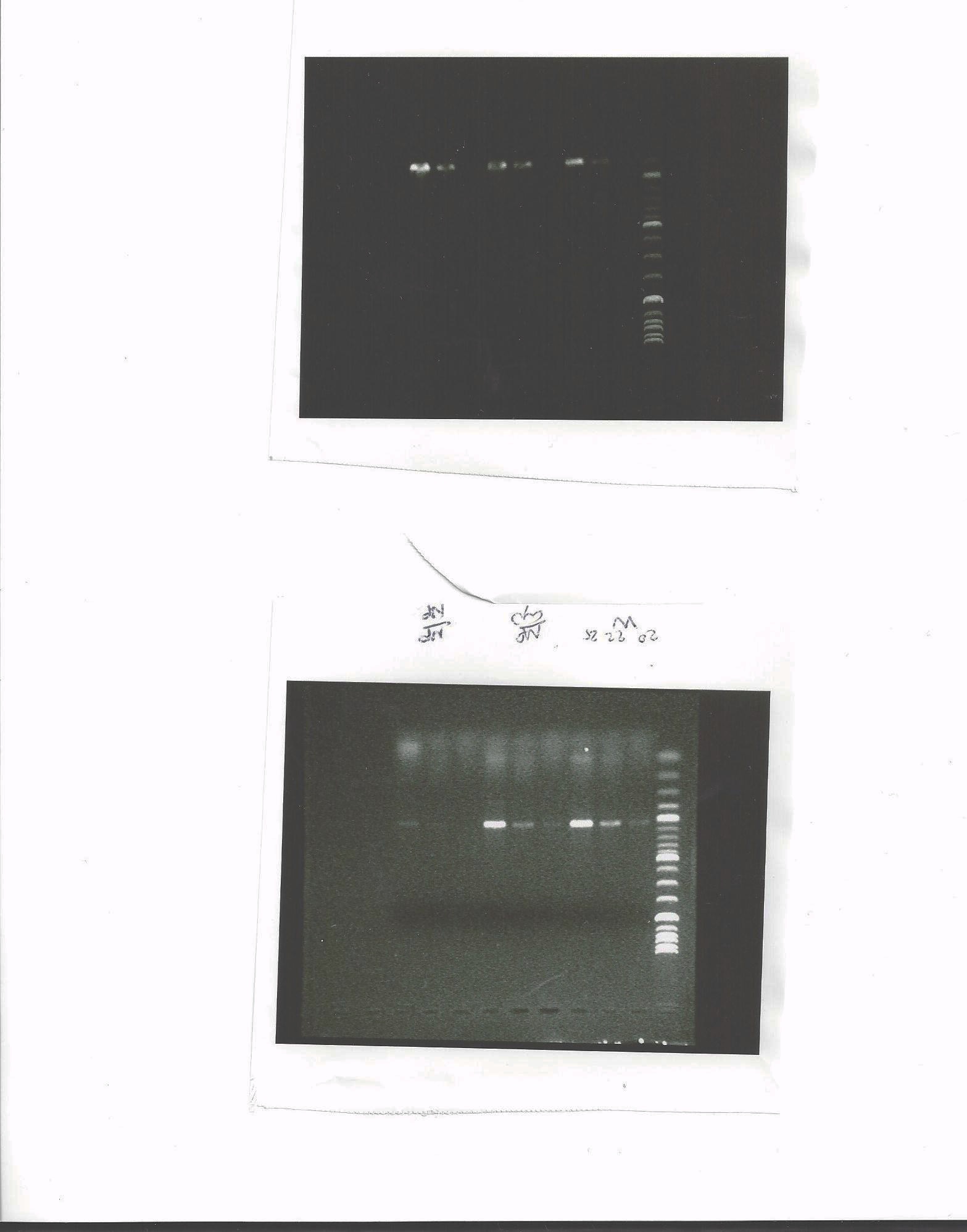

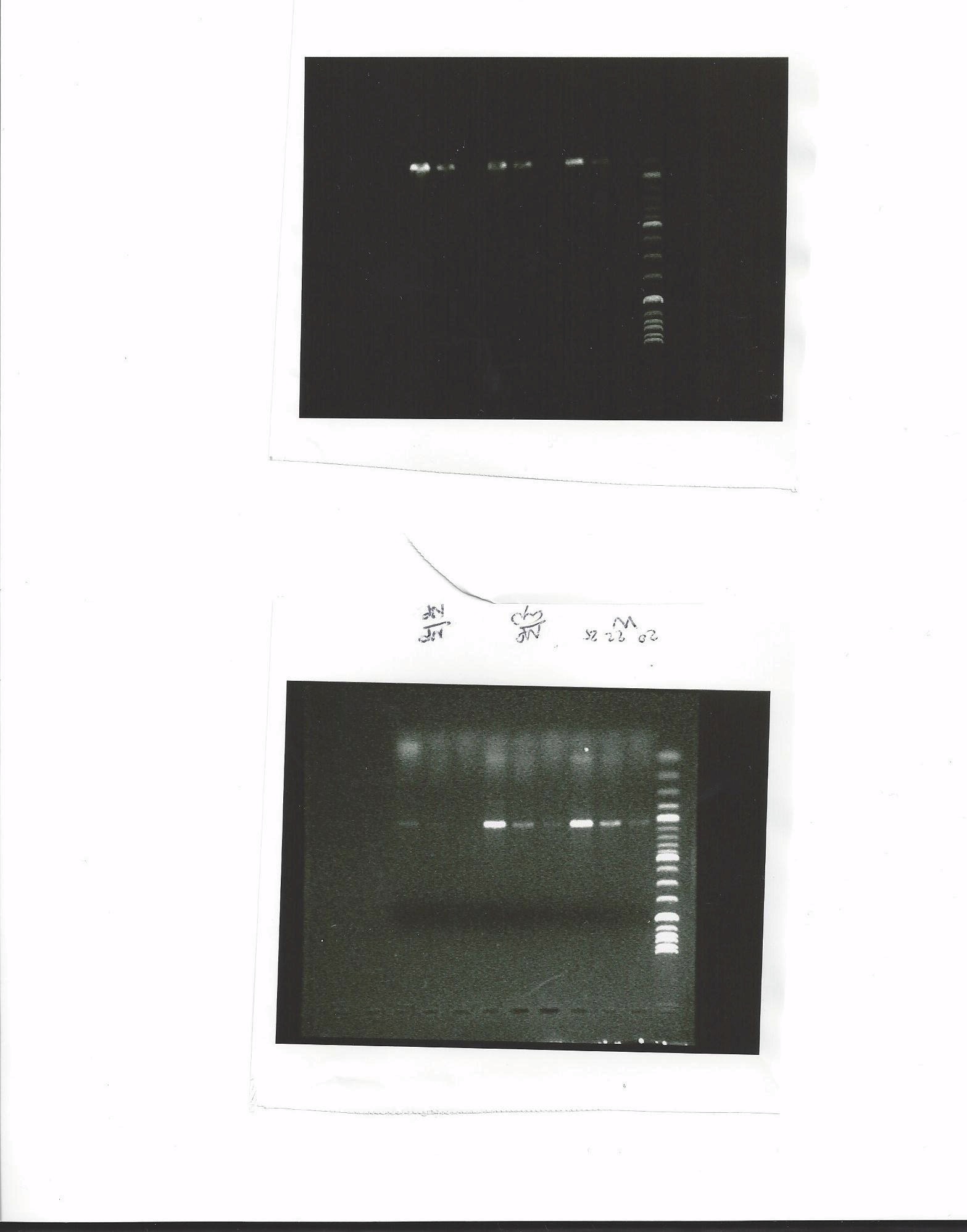


20 22 25 20 22 25 20 22 25

*rp49*

M

PCR cycles

*w1118*

*dadd1NP1240/+*

*dadd1NP1240/dadd1NP1240*


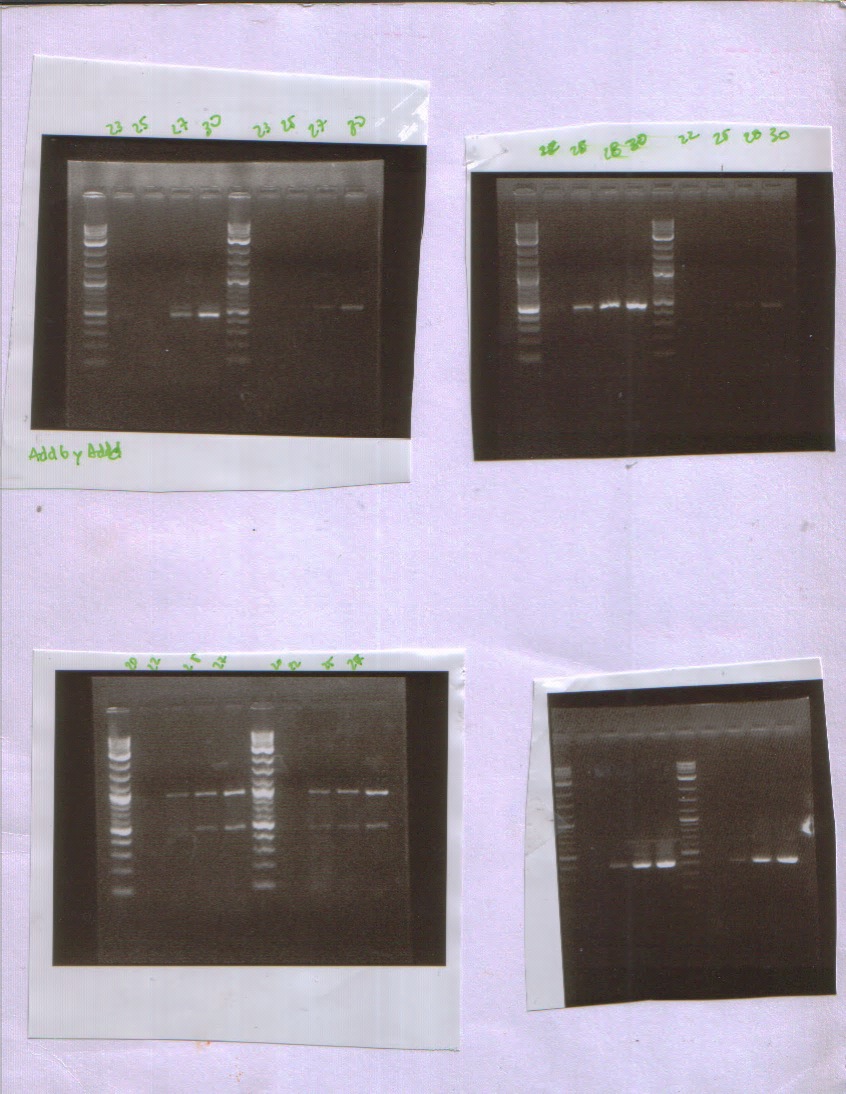

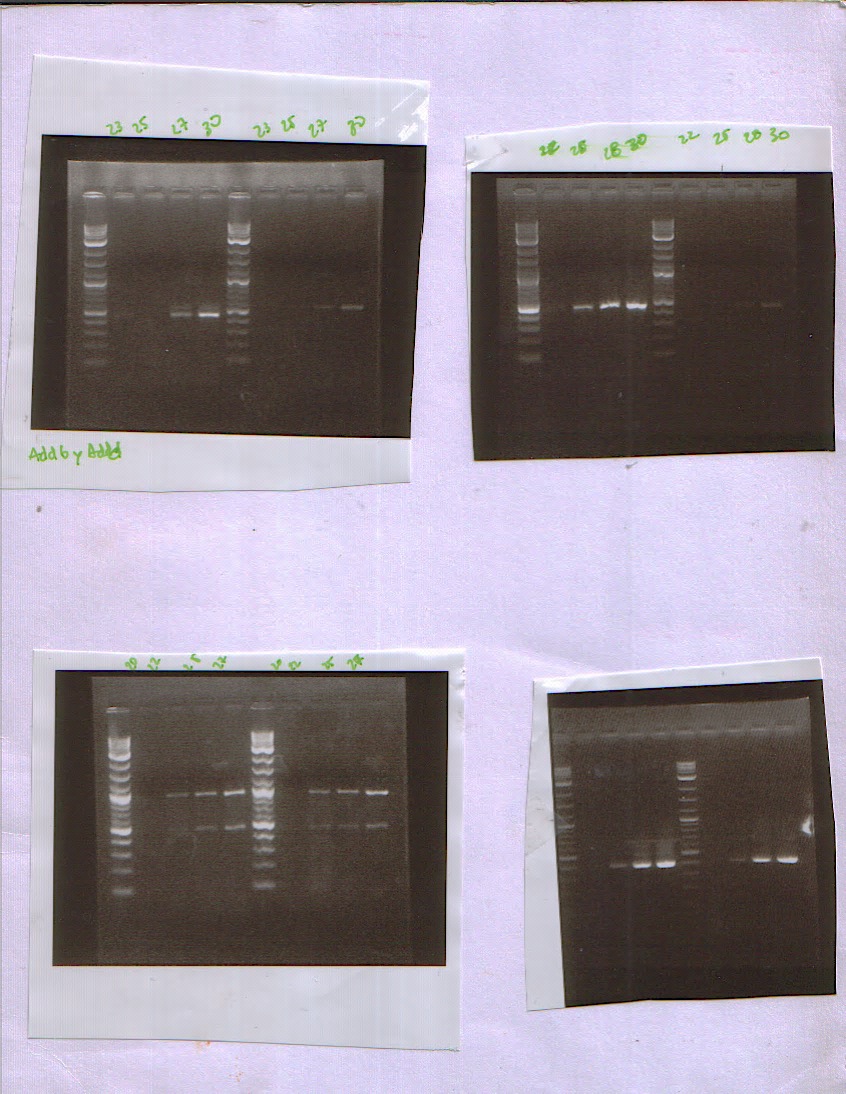


*rp49*

*dAdd1*

22 25 28 30 22 25 28 30

*w1118 dadd1NP0793/+*

M

Supplement: Figure S4 — Alleles dadd1NP1240 and dadd1NP0793 are hypomorphs. Semiquantitative RT-PCR from wild type and mutant dadd1 flies. dadd1 mRNA level is lower in the dadd1NP1240 (upper panel) and dadd1NP0793 (lower panel) heterozygous flies than in the dadd1 wild type flies (w1118). In homozygous dadd1NP1240/dadd1NP1240 (upper panel), dadd1 is even lower that in the heterozygous condition. rp49 transcript levels remained unchanged in the mutant alleles. (DOCX) [file pone.0113182.s004.docx]

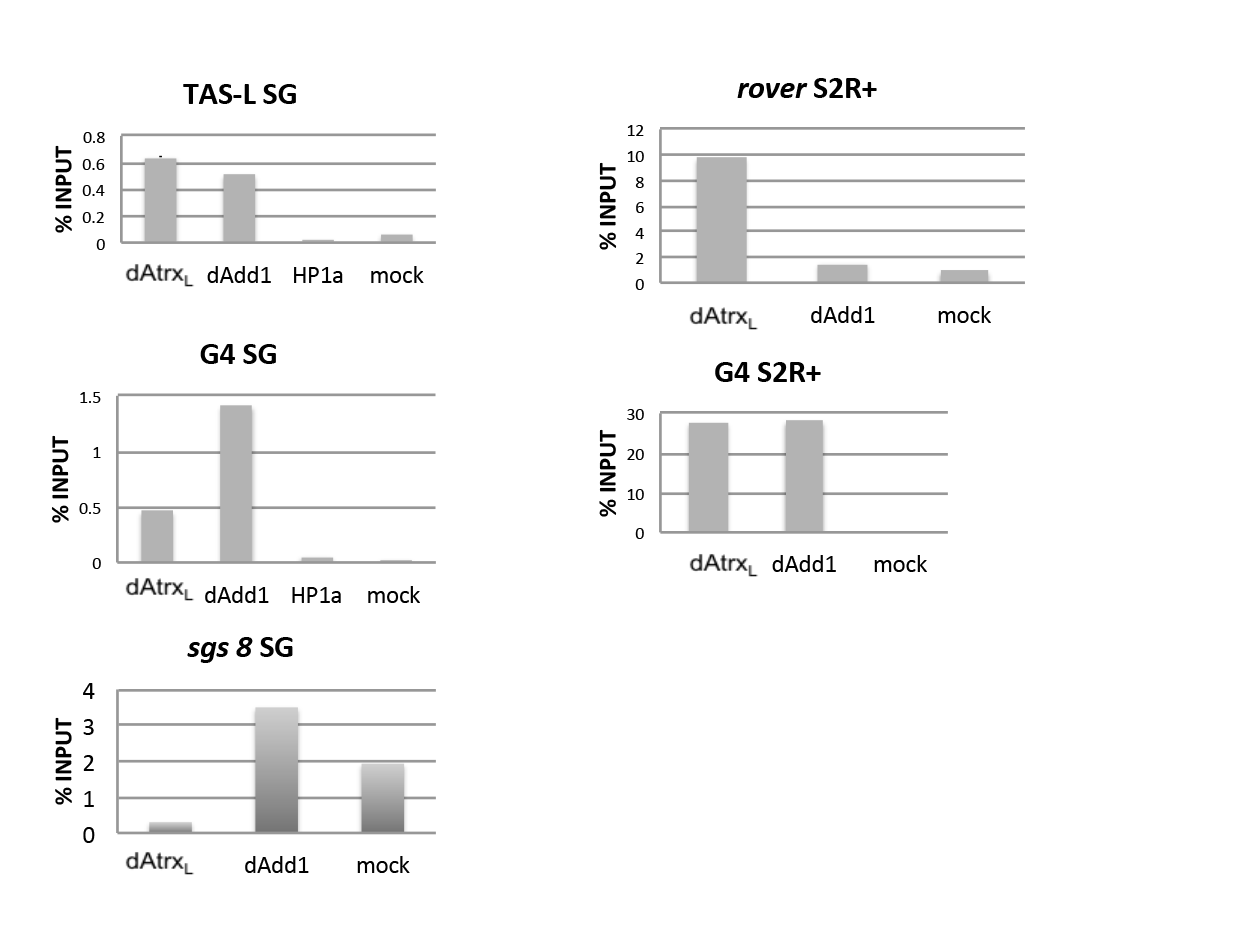

Supplement: Figure S5 — The dAdd1 proteins co-localize in vivo with dAtrxL and HP1a in some chromatin regions. ChIP assay using total extracts from third instar salivary glands (SG) prepared from wild-type larvae and S2R+ cells. Graphs represent the percentage of input precipitated using the different antibodies for the same regions. Note that in the rover region only dAtrxL is enriched. Three independent biological replicates were performed. (TIF) [file pone.0113182.s005.tif]
