# Supplementary material for: Characterization of the Drosophila Group Ortholog to the Amino-Terminus of the Alpha-Thalassemia and Mental Retardation X-Linked (ATRX) Vertebrate Protein
Source: PLoS One. 2014 Dec 1;9(12):e113182. doi: 10.1371/journal.pone.0113182 (PMC4249797; doi:10.1371/journal.pone.0113182)
Supplement: Supplementary file 2 [file pone.0113182.s002.docx]

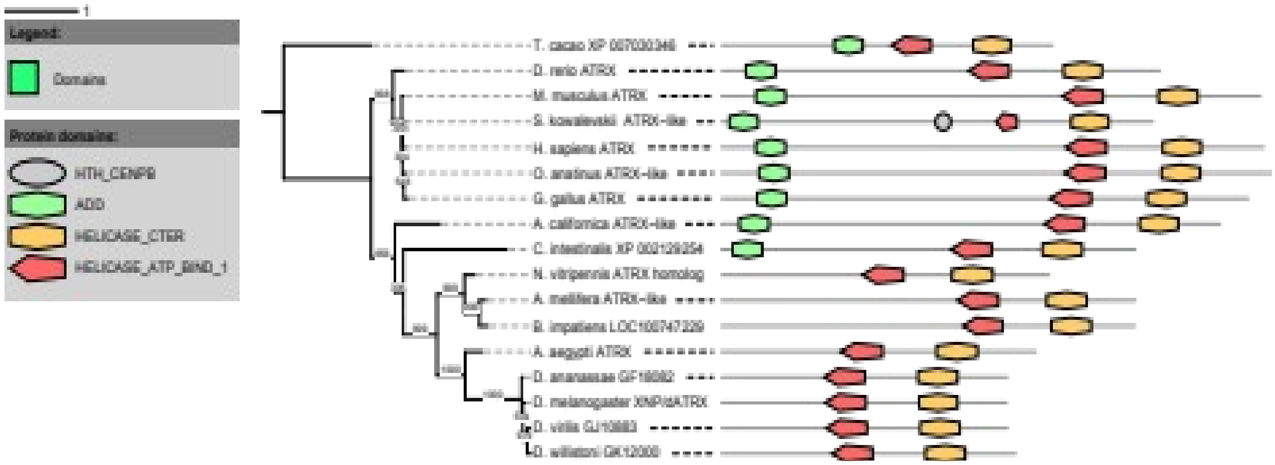
1
Legend:
Domains
Protein domains:
HTH_CENPB
ADD
HELICASE_CTER
HELICASE_ATP_BIND_1
T. cacao XP 007030346
D. rerio ATRX
M. musculus ATRX
S. kowalevskii ATRX-like
H. sapiens ATRX
O. anatinus ATRX-like
G. gallus ATRX
A. californica ATRX-like
C. intestinalis XP 002129254
N. vitripennis ATRX homolog
A. mellifera ATRX-like
B. impatiens LOC100747329
A. aegypti ATRX
D. ananassae GF18082
D. melanogaster XNP/dATRX
D. virilis GJ10883
D. willistoni GK12000
